# F245. COGNITIVE RESERVE DIFFERENCE IN AFFECTIVE AND NONAFFECTIVE PSYCHOSIS

**DOI:** 10.1093/schbul/sby017.776

**Published:** 2018-04-01

**Authors:** Silvia Amoretti, Bibiana Cabrera Llorca, Gisela Mezquida, Manuel J Cuesta, Mara Parellada, Ana Gonzalez-Pinto, Iluminada Corripio, Eduard Vieta, Miquel Bernardo

**Affiliations:** 1 Hospital Clinic of Barcelona, University of Barcelona; 2 Hospital Clinic Barcelona, Institut Clinic Neurociencies; 3 Complejo Hospitalario de Navarra, IdiSNA, Navarra Institute for Health Research; 4 Hospital General Universitario Gregorio Marañon, Universidad Complutense de;; 5 Hospital Universitario Araba, Universidad del País Vasco; 6 Hospital Santa Creu i St. Pau; 7 Institute of Neuroscience, Hospital Clinic, University of Barcelona

## Abstract

**Background:**

The cognitive reserve (CR) refers to the capacity of an adult brain to cope with pathology in order to minimize the symptoms (Stern, 2002). Recent studies have shown that CR is associated with clinical, functional and cognitive outcomes in patients with severe mental illness (de la Serna et al., 2013; Forcada et al., 2015; Anaya et al., 2016; Amoretti et al. ., 2016; Grande et al., 2017). Higher CR has been related to a later onset of psychosis, greater adherence and fewer psychotic symptoms (Barnett et al., 2006). However, there are no studies that evaluate longitudinally the role of CR depending on the diagnosis.

The objective is to analyze the impact of CR according to the diagnosis and to study whether having a high CR may be associated with better clinical, functional and cognitive outcomes.

**Methods:**

We gathered all the relevant clinical and sociodemographic data. All subjects were assessed clinically, neuropsychologically and functionally at baseline and after a two-year follow-up. To assess CR, three proxies have been integrated: premorbid IQ, years of education-occupation and leisure activities.

To determine whether the level of CR was associated with clinical, functional and neuropsychological outcomes and whether it was different between diagnoses, a multivariate analysis of variance was used.

**Results:**

285 DSM-IV patients with first episode of psychosis (FEP) were enrolled. The sample was divided into affective and non-affective groups.

In the non-affective group, those with high CR are older and had a better socioeconomic status, better functioning and cognitive performance and lower symptoms, as well as a shorter duration of untreated psychosis (DUP) and a later age of onset. After 2 years of follow-up, they showed significant differences in all the cognitive domains evaluated, except for the executive functions.

In the affective group, the patients with high and low CR showed differences in positive and manic symptoms, as well as in verbal memory at baseline. At 2 years of follow-up the differences were observed in functionality, positive and negative symptoms and in verbal memory. There were no significant differences in terms of age, gender, DUP, or age of onset, although significant differences were found in socioeconomic level (p = 0.038).

**Discussion:**

Higher CR can result in better recovery and functioning and in higher cognitive performance in patients with a FEP. Therefore, we propose that early interventions focused on the promotion of neuropsychological abilities and CR could reduce the harmful impact of this disease.

However, it is necessary that these interventions should be personalized taking into account that CR plays a differential role according to the diagnosis.

